# Selected problems with boron determination in water treatment processes. Part I: comparison of the reference methods for ICP-MS and ICP-OES determinations

**DOI:** 10.1007/s11356-016-6328-7

**Published:** 2016-03-04

**Authors:** Ewa Kmiecik, Barbara Tomaszewska, Katarzyna Wątor, Michał Bodzek

**Affiliations:** AGH University of Science and Technology, Faculty of Geology, Geophysics and Environmental Protection, Mickiewicza 30 Av, 30-059 Kraków, Poland; Mineral and Energy Economy Research Institute, Polish Academy of Sciences, Wybickiego 7, 31-261 Kraków, Poland; Silesian University of Technology, Institute of Water and Wastewater Engineering, Konarskiego 18, 44-100 Gliwice, Poland; Institute of Environmental Engineering of the Polish Academy of Sciences, M. Curie-Skłodowskiej 34, 41-819 Zabrze, Poland

**Keywords:** Boron, Analytical determination, Desalination, Geothermal water, Reverse osmosis

## Abstract

The aim of the study was to compare the two reference methods for the determination of boron in water samples and further assess the impact of the method of preparation of samples for analysis on the results obtained. Samples were collected during different desalination processes, ultrafiltration and the double reverse osmosis system, connected in series. From each point, samples were prepared in four different ways: the first was filtered (through a membrane filter of 0.45 μm) and acidified (using 1 mL ultrapure nitric acid for each 100 mL of samples) (FA), the second was unfiltered and not acidified (UFNA), the third was filtered but not acidified (FNA), and finally, the fourth was unfiltered but acidified (UFA). All samples were analysed using two analytical methods: inductively coupled plasma mass spectrometry (ICP-MS) and inductively coupled plasma optical emission spectrometry (ICP-OES). The results obtained were compared and correlated, and the differences between them were studied. The results show that there are statistically significant differences between the concentrations obtained using the ICP-MS and ICP-OES techniques regardless of the methods of sampling preparation (sample filtration and preservation). Finally, both the ICP-MS and ICP-OES methods can be used for determination of the boron concentration in water. The differences in the boron concentrations obtained using these two methods can be caused by several high-level concentrations in selected whole-water digestates and some matrix effects. Higher concentrations of iron (from 1 to 20 mg/L) than chromium (0.02–1 mg/L) in the samples analysed can influence boron determination. When iron concentrations are high, we can observe the emission spectrum as a double joined and overlapping peak.

## Introduction

Borates are widely found in nature, and they are present in oceans, sedimentary rocks, soil, coal and shale. Naturally occurring boron is found in groundwater, primarily as a result of leaching from rocks and soils that contain borates and borosilicates. Boron is detected in surface water and groundwater in various locations around the world, including sea and river waters, where it mainly occurs in the form of boric acid.

Boron compounds are also used during manufacturing processes, e.g. glass production, fibreglass, borosilicate glass, agricultural fertilisers and herbicides, laundry bleach, soaps and detergents, flame retardants and many other processes (Dill 2010). These compounds are often encountered in natural and waste waters where their concentration may reach tens and hundreds of milligrams per 1 dm^3^ (Öner et al. 2011). Hence, the borate contents of surface and groundwater can be increased as a result of wastewater discharges (WHO 2011), especially from municipal (Motyka et al. 2005; Tomaszewska 2009), power plant (Tomaszewska 2009) or chemical landfill leachates (Malina 2004; Turek et al. 2007; Witkowski et al. 2008). As a result, boron is a commonly known contaminant of drinking water that affects the reproductive ability of living organisms.

Therefore, there exists the need to remove this element from the aqueous environment. Actually, many materials have been tested for this purpose including fly ash, zeolite and active carbons (Polat et al. 2004; Yüksel and Yürüm 2010). Zeolites seem to be promising materials, which, due to their ion-exchange properties and molecular sieve, have already been used in other fields of engineering and environmental protection, including removing heavy metals and ammonium ions from waste water or treating mine water with radioactive elements (Franus and Wdowin 2010; Liu et al. 2010; Niu et al. 2012; Merrikhpour and Jalali 2013; Chałupnik et al. 2013; Franus et al. 2014). A process that is more and more frequently used in the synthesis of zeolites is the treatment of fly ash with sodium hydroxide (Querol et al. 2001; Franus et al. 2014; Wdowin et al. 2014).

The level of boron concentration in drinking water varies widely and depends on the source of the drinking water, but for most of the world, the range is judged to be between 0.1 and 0.3 mg/L (Öner et al. 2011). In seawater, the boron concentration ranges from 0.5 to 9.6 mg/L, an average value is found between 4.5 ppm and 4.6 mg/L (Woods 1994; Farhat et al. 2013; Kabay et al. 2010). A high boron concentration is also a common feature of geothermal water sources (Dill 2010; Bundschuh et al. 2013; Tomaszewska and Szczepański 2014; Tomaszewska et al. 2014), especially when TDS is greater than 1 g/L (Tomaszewska and Bodzek 2013a).

Due to its interaction with the environment, the boron concentration in drinking water is strictly controlled according to the WHO (up to 2.4 mg/L) and European Union regulations (up to 1.0 mg/L). In the case of water discharged to the environment, the boron level has to be limited to 1.0 mg/L (Polish government regulations). The World Health Organization had for many years recommended that the limits of boron concentration in drinking water be 0.5 mg/L. The new guideline value is based on a human health perspective (WHO 2011), which was revised by the WHO Drinking-Water Quality Committee, acting on reports from the latest studies published in the USA (WHO 2011).

In many parts of the world, the presence of boron prevents the direct use of water for irrigation or as potable water and causes chemical pollution and environmental problems in ground and surface waters (Dill 2010). Boron is also an essential micronutrient for plants, but the concentration level required for optimum growth differs between plant species. In effect, there is a narrow margin between boron deficiency and toxicity in some plants (Kot 2009; Yavuz et al. 2013; Wolska and Bryjak 2013; Tomaszewska and Bodzek 2013a, b; Bodzek 2015). For many reverse osmosis (RO) plants producing drinking water from seawater or brackish water, it is challenging to reduce boron concentrations down to the stringent limits specified in the new regulations. The boron problem is more critical in many geothermal waters since they have higher boron concentrations than those in seawater.

On the other hand, it has been proved that boron is an essential element in the human diet; however, its specific biochemical function has not yet been identified (WHO 2011; Kabay et al. 2010). Between the middle of nineteenth century and the beginning of the twentieth century, boron compounds were used for treating various medical conditions including epilepsy, malaria, urinary tract infections and exudative pleuritis (EPA 2008). Culver and Hubbard (1996) report on early cases of boron treatment for epilepsy and on dose values ranging from 2.5 to 24.8 mg B/kg-day being prescribed for many years (Culver and Hubbard 1996). Overall, more than half of the average total exposure to boron comes from the diet. Previous studies have concluded that boron is important in the metabolism and the utilisation of calcium for the human bone structure (Mr and Samman 1993). Other benefits of boron include the improvement of brain function, the psychomotor response and the response to oestrogen injections in postmenopausal female patients. A crucial role of boron in keeping bones and joints healthy has also been described, and it has been effective in treating various forms of arthritis (Newnham 1994; Kabay et al. 2010). The guidance presented by WHO (2011) claims that short- and long-term exposure of the breathing system (exposure of respiratory paths, especially the pharynx) to boric acid or borax in laboratory animals has demonstrated that the male reproductive tract is a consistent target of toxicity (WHO 2011). Testicular lesions have been observed in rats, mice and dogs that were given boric acid or borax in food or drinking water. Developmental toxicity has been demonstrated experimentally in rats, mice and rabbits. Negative results in a large number of mutagenicity assays indicate that boric acid and borax are not genotoxic. In long-term studies in mice and rats, boric acid and borax caused no increase in tumour incidence (WHO 2011).

In that context, assessment of water quality and the analytical precision of boron determination plays a special role in the treatment of sea, ocean or geothermal water. It indeed determines the proper selection of the treatment process, which allows for the effective reduction of boron concentration to the level specified in the relevant legislation. The detection and quantification of boron and its stable isotopes has been conducted using several different techniques reported in the literature (Sah and Brown 1997; Witczak et al. 2007; Kot 2009; Witczak et al. 2013; Farhat et al. 2013). These techniques are presented in Table 1.

**Table 1:** Principles of the major boron analysis techniques

Technique		Principles of the technique
Plasma based	ICP-OES	Formation of free atoms of boron then monitors the wavelength emissions from boron excited atoms at its corresponding wavelengths
	ICP-MS	Ionizing boron into B^+^ ions, then measuring B isotopes abundance based on their mass-to-charge ratio (*m*/*z* 10 and 11)
TIMS	PTIMS	Converting boron into alkali or metal metaborate cations such as Na_2_BO_2_^+^ (*m*/*z* 88 and 89) and Cs_2_BO_2_^+^ (*m*/*z* 308 and 309) then measuring their corresponding *m*/*z* ratio
	NTIMS	Converting boron into metaborate anions of BO_2_^−^ (*m*/*z* 42 and 43) then measuring their corresponding *m*/*z* ratio
Non-MS-based	Spectrophotometry	Adding specific reagents to the boron samples for colour development, then measuring the absorbance at wavelengths, respective of the reagent (e.g. curcumin or carmine method—Standard methods, 2012)
	Nuclear	Bombarding boron with neutrons causing the production of a-particles and g-particles that are monitored to measure ^10^B isotope abundance

After Farhat et al. (2013)

*m*/*z* mass-to-charge ratio

This manuscript presents two plasma based methods mentioned in Table 1. The ICP methods are often used for boron determination in water samples. In this method, samples containing boron compounds are atomised into elemental B and ionised into B^+^ cations. In these forms, boron is analysed using different types of detector (Farhat et al. 2013). The most popular are inductively coupled plasma optical emission spectrometry (ICP-OES) and inductively coupled plasma mass spectrometry (ICP-MS). Both ICP-MS and ICP-OES methods are published in the appropriate standards PN-EN ISO 11885:2009 and PN-EN ISO 17294–1:2007 and provide the possibility of analysing boron concentration over a wide range. The problems with boron analysis using these two methods are widely described in the literature (Dulski 1996; Al-Ammar et al. 1999; Aggerwal et al. 2003; Parks 2005; Eppich et al. 2011). The biggest problem is memory but sample matrix composition could also affect the bias and variability of the ICP-MS method. Research provided by Garbarino (1999) shows that matrix composition can suppress the ionisation efficiency of the plasma and result in negatively biased results from analysis. This effect can be significant for whole-water matrices because of the level of dissolved solid concentrations. Analysis of water samples performed by Garbarino (2000) indicates that differences in boron concentrations obtained by the ICP-MS and ICP-OES methods are significant, and they are caused by several high-level concentrations in selected whole-water digestates.

In the ICP-OES method, the boron concentration is detected on the basis of the value of the electromagnetic radiation emitted by excited B atoms. Typically monitored wavelengths characteristic for boron are 182.52, 249.678 and 249.773 nm. Detection limits vary from 0.005 to 0.01 mg/L. The common interferences are from iron and chromium (van de Wiel 2003).

The ICP-MS methods allow the analysis of two boron isotopes: ^10^B and ^11^B. Concentrations of boron isotopes are determined on the basis of their mass-to-charge (*m*/*z*) ratio. The detection limit is 0.01 mg/L. There are no spectroscopic interferences (van de Wiel 2003). The very important factors in boron determination with the ICP-MS method are sample matrices, memory effects and some instrument parameters (Sah and Brown 1998).

The most common method of sample preparation for ICP is filtration and acidification with a proportion of 1 mL of concentrated HNO_3_ per 100 mL of water sample. So, in this situation, only dissolved boron is analysed.

## Objectives

Proper collection of water samples, fixation and transport to the laboratory are especially important matters in water sampling when choosing the appropriate analytical procedure. The main objective of this study was to determine the precision of analytical determination of boron in water samples which have been taken during geothermal water treatment processes. The paper presents a comparison of the ICP-MS and ICP-OES reference method determinations related to boron quantification during water treatment.

## Materials and methods

A total of 56 samples were collected during different desalination processes including ultrafiltration and double reverse osmosis systems connected in series (Tomaszewska and Bodzek 2013b). Four samples from each point in the treatment system were taken for determination using ICP-OES and ICP-MS methods according to the requirements of the PN-ISO 5667–11:2004 standard. Samples were prepared in different ways, using four sampling protocols:

1. Fourteen samples were filtered (through a membrane filter of 0.45 μm) and acidified (using 1 mL ultrapure nitric acid for each 100 mL of sample) (FA)
2. Fourteen samples were unfiltered and not acidified (UFNA)
3. Fourteensamples were filtered but not acidified (FNA)
4. Fourteen samples were unfiltered but acidified (UFA)

Samples were collected in polyethylene bottles. Then, samples were cooled and sent immediately to the laboratory where only plastic bottles and probes were used to avoid sample contamination, for example from borosilicate glass.

To treat how big an influence filtration and acidification has on the boron concentration in water samples, the authors also compare the results obtained for certified samples with known boron concentration. The analysis was performed at three concentration levels and repeated five times:

- 2.5 mg B/L
- 1.0 mg B/L
- 0.5 mg B/L

The samples were analysed using ICP-OES (Optima 7300DV - PN-EN ISO 11885:2009) and ICP-MS methods (ELAN 6100 - PN-EN ISO 17294–1:2007, PN-EN ISO 17294–2:2006). The certified ranges for the boron determination, declared by the laboratory, are as follows: for ICP-MS, 0.01–100 mg/L; for ICP-OES, 0.1–100 mg/L (PCA certificate, no AB 1050), and the uncertainty of boron determination is lower than 20 % with both methods.

The Optima 7300DV Inductively Coupled Plasma Optical Emission Spectrometer (ICP-OES) is a dual detector system. The UV detector covers an extended ultraviolet wavelength range from 165 to 403 nm whereas the VIS detector covers the visible wavelength range from 404 to 782 nm. In dual-view instruments plasma can be viewed either axially or radially. The Sample Introduction Compartment with Quick Change Torch Module characteristics and some apparatus parameters are shown in Table 2.

**Table 2:** Sample introduction compartment characteristics and some ICP-OES spectrometer parameters for boron determination in water samples

Sample introduction compartment/parameter	Type/value
Torch	Standard alumina injector with a 2.0-mm inner diameter
Spray chamber	Double-pass Scott-type
Nebuliser	The Gem Tip cross flow, pneumatic
RF frequency	40 MHz
RF generator	1300 W
Plasma flow	15 L/min
Auxiliary flow	0.2 L/min
nebuliser flow	0.8 L/min
Sample flow rate	1.5 mL/min
Equilibration time	30 s
Wavelength	249.678 nm
Interferences	Fe, Cr
Replicates	3

The ELAN 6100 Inductively Coupled Plasma Mass Spectrometer (ICP-MS) has an original dual-stage detection system with a discrete dynode electron multiplier. The Sample Introduction Compartment characteristics and some apparatus parameters are shown in Table 3.

**Table 3:** Sample introduction compartment characteristics and some ICP-MS spectrometer parameters for boron determination in water samples

Sample introduction compartment/parameter	Type/value
Torch	Quartz
Spray chamber	Double-pass Scott-type
Nebuliser	Cross flow
RF frequency	40.16 MHz
RF generator	1050 W
Plasma flow	15 L/min
Auxiliary flow	1.5 L/min
Nebuliser flow	0.93 L/min
Sample flow rate	1.5 mL/min
Monitored isotopes	^11^B
Interferences	No interferences
Internal standard	^89^Y
Equilibration time	400–3000 ms
Replicates	3

All analyses were performed in the certified Hydrogeochemical Laboratory of the Hydrogeology and Engineering Geology Department of the University of Science and Technology in Cracow (PCA certificate, no AB 1050). The laboratory takes part in proficiency testing and interlaboratory comparisons obtaining satisfactory results (Z-score absolute value lower than 2). In this laboratory, an internal quality control system is also implemented.

## Results and discussion

The most commonly used methods of boron determination in Polish laboratories are ICP-MS and ICP-OES which form the methods of reference (Witczak et al. 2013). ICP is a type of plasma source formed from electric currents that are caused by electromagnetic induction in a rarefied gas such as argon. Samples are usually prepared in the aqueous phase using steps involving extraction and purification and are then introduced into the plasma of the instrument via a nebuliser and spray chamber (Sah and Brown 1997; Kmiecik and Podgórni 2009; Kmiecik 2011). ICP-OES, earlier known as ICP atomic emission spectroscopy (ICP-AES), is one type of ICP that detects electromagnetic radiation emitted from energised atoms and ions produced by the plasma source in which the wavelength of the radiation emitted is characteristic of an element (Sah and Brown 1997). The coupling of ICP with a mass spectral detector (ICP-MS) allows boron determination with the simultaneous measurement of boron concentration and its isotopic abundance (^11^B and ^10^B) leading to lower detection limits and higher sensitivity (Sah and Brown 1997; Farhat et al. 2013). Instead of monitoring the wavelength-specific emissions of the energised ions as in ICP-OES, the ICP-MS method measures the ions based on their mass-to charge (*m*/*z*) ratio; thus, it simultaneously measures boron concentration and its stable isotope abundance.

The results of the analysis for the control samples, blank samples and certified reference materials prove that the analysis of boron concentration in certified ranges (for ICP-MS from 0.01 to 100 mg/L and for ICP-OES from 0.1 to 100 mg/L) gives appropriate results.

The precision of boron determination using both methods is lower than 20 % (estimated as RSD value) whereas accuracy varies from 80 to 120 %. An estimate was made of the expanded uncertainty of both the ICP-MS and the ISP-OES methods and did not exceed 20 %.

Table 4 summarises the basic descriptive statistics of the results obtained by both methods and using samples prepared differently for each of the analyses.

**Table 4:** Boron concentration in the water samples analysed (descriptive statistics [mg/L])

Sampling protocol	Range	Minimum	Maximum	Mean
ICP-MS method				
FA	3.01	0.18	3.19	1.19
FNA	3.58	0.18	3.76	1.28
UFA	3.12	0.17	3.29	1.18
UFNA	3.68	0.17	3.85	1.29
ICP-OES method				
FA	6.46	0.29	6.75	2.04
FNA	6.34	0.25	6.59	2.00
UFA	6.53	0.26	6.79	2.04
UFNA	6.53	0.24	6.77	2.02

*FA* filtered but acidified, *FNA* filtered but not acidified, *UFA* unfiltered but acidified, *UFNA* unfiltered but not acidified

The mean values of the results obtained by ICP-OES are higher than the means of the results obtained by ICP-MS. Also, the ranges of concentrations of boron obtained by ICP-OES in the samples analysed are greater than those obtained using the ICP-MS method regardless of the method of preparing samples for analysis (Table 4). These differences might be caused by several high-level concentrations in selected whole-water digestates and some matrix effects in the water samples analysed. The research showed that a greater concentration of iron, in concentrations from 1 to 20 mg/L, than chromium (0.02–1 mg/L) in the water samples analysed can influence boron determination. When iron concentrations are high, we can observe the emission spectrum as double joined with an overlapping peak. This is compatible with the results of a comparison of boron concentrations performed by the Instituto di Geoscienze e Georisorse Area di Ricerca del CNR and International Atomic Energy Agency (IAEA), Vienna, Austria (Gonfiantini et al. 2003).

**Fig. 1:**
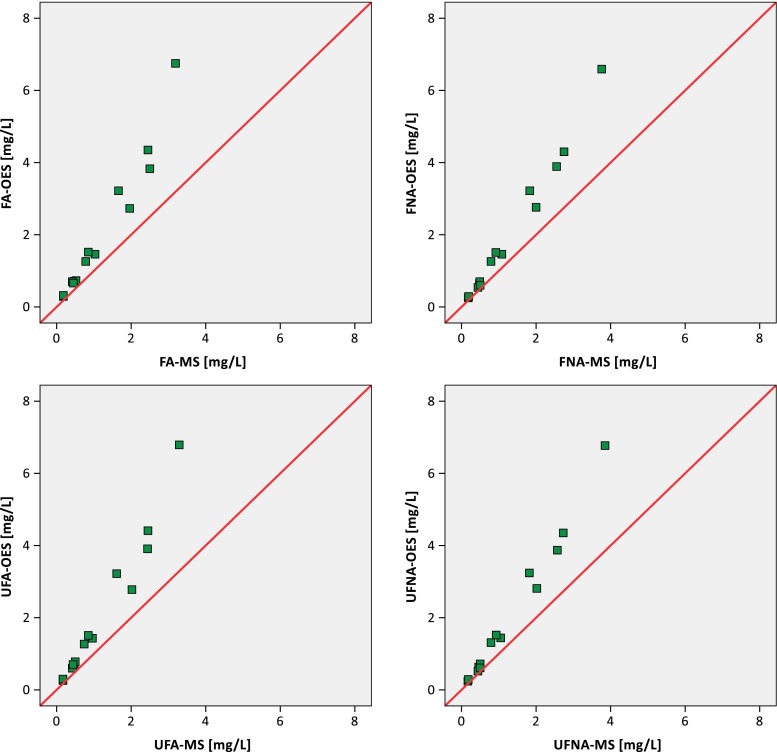
Boron concentrations in the water samples analysed—comparison of four sampling protocols and two methods of analysis

The results of the boron determination in the water samples tested are also presented on scatterplots (Fig. 1).

The graphs show that the results of boron determinations obtained by both methods have a strong linear correlation. This is confirmed by high values of Pearson correlation coefficients (Table 5). These correlations are statistically significant at *p* < 0.05, they are not dependent on the method of sample preparation (sampling protocol).

**Table 5:** Boron concentration in the water samples analysed (correlation of paired samples)

Sampling protocol/methods of boron determination	Number	Correlation coefficient	Significance
FA-MS [mg/L] and FA-OES [mg/L]	14	0.977	0.000
FNA-MS [mg/L] and FNA-OES [mg/L]	14	0.993	0.000
UFA-MS [mg/L] and UFA-OES [mg/L]	14	0.981	0.000
UFNA-MS [mg/L] and UFNA-OES [mg/L]	14	0.993	0.000

*FA* filtered but acidified, *FNA* filtered but not acidified, *UFA* unfiltered but acidified, *UFNA* unfiltered but not acidified

For boron concentrations less than 1 mg/L, there is a high consistency seen in the results (the points on the graphs are arranged on the diagonal; Fig. 1). At higher concentrations of boron, higher values of the results are achieved in the samples analysed using ICP-OES than in those using ICP-MS.

In order to verify whether the mean values of boron concentration obtained by both methods are statistically significant, a *t* test was performed for dependent samples (using IBM SPSS v. 21 software). Table 6 summarises the results of this test.

**Table 6:** Boron concentration in the water samples analysed (paired samples test)

Sampling protocol/methods of boron determination	Paired differences					*t*	*df*	Significance (two-tailed)
	Mean	Std. dev.	Std. error mean	95 % Confidence interval of the difference				
				Lower	Upper			
FA-MS [mg/L] and FA-OES [mg/L]	−0.85	0.965	0.258	−1.4063	−0.2922	−3.294	13	0.006
FNA-MS [mg/L] and FNA-OES [mg/L]	−0.72	0.799	0.214	−1.1802	−0.2569	−3.363	13	0.005
UFA-MS [mg/L] and UFA-OES [mg/L]	−0.86	0.967	0.258	−1.4219	−0.3051	−3.341	13	0.005
UFNA-MS [mg/L] and UFNA-OES [mg/L]	−0.74	0.821	0.219	−1.2107	−0.2621	−3.354	13	0.005

*FA* filtered but acidified, *FNA* filtered but not acidified, *UFA* unfiltered but acidified, *UFNA* unfiltered but not acidified

The *Mean* column displays the average difference between the measurements obtained with the two analytical methods. The *Standard deviation* column displays the standard deviation of the average difference score. The *Standard error mean* column provides an index of the variability one can expect in repeated random samples of 14 water samples. The *95 % Confidence interval of the difference* provides an estimate of the boundaries between which the true mean difference lies in 95 % of all possible random samples of 14 water samples similar to those analysed. The *t* statistic is obtained by dividing the mean difference by its standard error; *df* means degrees of freedom. The *Significance (two-tailed)* column displays the probability of obtaining a *t* statistic whose absolute value is equal to or greater than the *t* statistic obtained.

The table shows that the means of the results obtained by ICP-MS are lower by 0.72–0.86 mg/L than those obtained by ICP-OES. The means of results obtained by ICP-MS are significantly lower than those obtained by ICP-OES, regardless of the method of sample preparation for analysis (in all cases, significance <0.05; Table 6).

When analysis is performed within 24 h of sample collection, —sample filtration and proper preservation is very important. On the basis of the differences presented in Table 7, we can estimate the impact of filtration and acidification on the boron determinations in the water samples analysed using the two methods.

**Table 7:** Impact of filtration and acidification on boron determination in the water samples analysed—differences between the results [mg/L]

Sampling protocol	Sample													
	1	2	3	4	5	6	7	8	9	10	11	12	13	14
ICP-MS method														
FA–FNA	−0.06	0.00	−0.04	−0.01	0.03	−0.05	−0.01	−0.06	−0.01	−0.17	−0.29	−0.56	−0.03	−0.05
UFA–UFNA	−0.09	0.00	0.00	0.01	0.00	−0.13	−0.05	−0.08	−0.01	−0.21	−0.28	−0.56	−0.02	−0.06
FA–UFA	0.06	0.01	−0.06	0.00	0.02	0.06	0.04	0.00	0.00	0.05	0.00	−0.10	−0.01	0.01
FNA–UFNA	0.03	0.01	−0.02	0.02	0.00	−0.02	0.00	−0.01	0.01	0.01	0.01	−0.10	0.00	0.00
ICP-OES method														
FA–FNA	0.06	0.04	0.02	0.07	0.01	0.00	0.00	0.07	0.05	0.17	0.35	0.72	0.19	0.11
UFA–UFNA	0.08	0.02	−0.03	0.02	0.06	0.17	0.01	0.07	0.02	0.19	0.34	0.59	0.10	0.16
FA–UFA	−0.03	0.02	0.01	0.05	−0.07	−0.13	−0.06	0.01	0.02	−0.05	−0.06	0.05	0.11	−0.05
FNA–UFNA	−0.01	0.00	−0.03	0.00	−0.02	0.04	−0.05	0.01	−0.01	−0.03	−0.07	−0.08	0.02	−0.01

*FA* filtered but acidified, *FNA* filtered but not acidified, *UFA* unfiltered but acidified, *UFNA* unfiltered but not acidified

For the ICP-MS method, we can observe that the boron concentration in acidified samples is minutely lower than in those nonacidified samples whereas the results for filtered samples are generally greater than for unfiltered samples. The opposite situation can be observed with the ICP-OES method. The results for acidified samples are greater than for the nonacidified water samples whereas filtration caused a decline in boron concentrations in the same water samples (Table 7).

To analyse boron behaviour under different conditions (after filtration and/or acidification), an analysis of samples with known boron concentration was performed. These samples contain boron at three concentration levels: 0.5, 1.0 and 2.5 mg/L.

The results of the comparison of the four sampling protocols tested with average boron concentrations in certified samples are shown in Table 8.

**Table 8:** Average boron concentrations in the certified samples [mg/L]

Sampling protocol	Average boron concentration in certified sample [mg/L]		
	0.5 mg/L standard	1 mg/L standard	2.5 mg/L standard
ICP-MS method			
FA	0.495	0.997	2.662
UFA	0.505	0.985	2.688
FNA	0.479	0.934	2.477
UFNA	0.486	1.019	2.486
ICP-OES method			
FA	0.488	0.904	2.710
UFA	0.494	0.994	2.665
FNA	0.481	0.973	2.569
UFNA	0.503	0.955	2.525

*FA* filtered but acidified, *FNA* filtered but not acidified, *UFA* unfiltered but acidified, *UFNA* unfiltered but not acidified

**Fig. 2:**
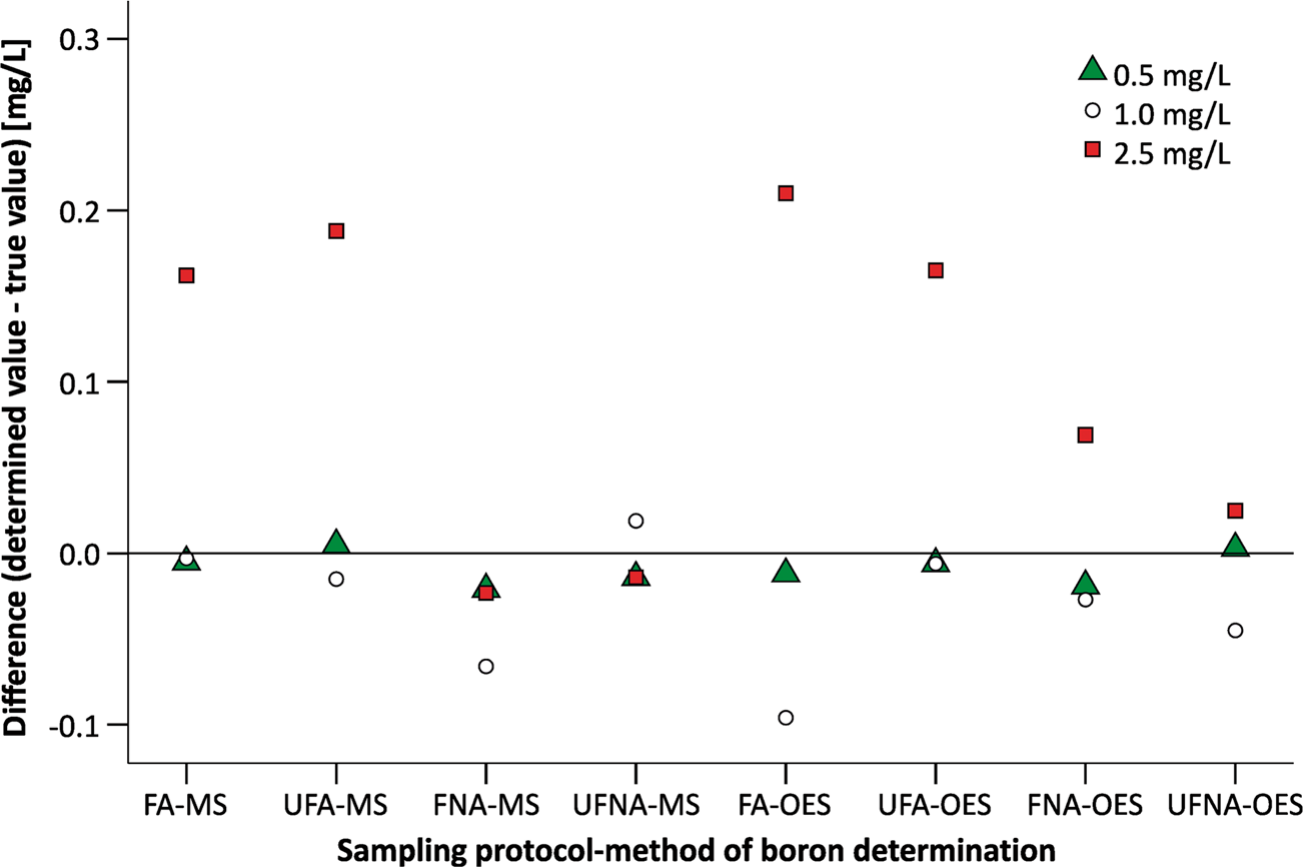
Differences between results obtained during analysis and the true values of boron concentrations in the standard sample—comparison of four sampling protocols and two methods of analysis

The results of the study show that sample filtration and acidification generally causes a decrease in boron concentration with low concentrations of boron (0.5 and 1.0 mg B/L) whereas in the case of higher concentrations (2.5 mg B/L), both filtration and acidification influence the increase in boron concentration in the standard examples analysed. These relations are not dependent on the method of analysis (Fig. 2, Table 8).

In order to verify if the mean values of boron concentration in standard samples obtained by different methods and using different sampling protocols are statistically significant, a *t* test for dependent samples was performed. Table 9 provides a summary of the results of this test.

**Table 9:** Boron concentrations in the certified samples (paired samples test)

Sampling protocol/methods of boron determination	Paired differences					*t*	*df*	Significance (two-tailed)
	Mean	Std. deviation	Std. error mean	95 % Confidence interval of the difference				
				Lower	Upper			
0.5 FA-MS–0.5 FA-OES	0.007	0.004	0.002	0.002	0.011	3.949	4	0.017
0.5 FNA-MS–0.5 FNA-OES	−0.003	0.006	0.002	−0.010	0.004	−1.162	4	0.310
0.5 UFA-MS–0.5 UFA-OES	0.011	0.005	0.002	0.004	0.017	4.504	4	0.011
0.5 UFNA-MS–0.5 UFNA-OES	−0.016	0.010	0.004	−0.028	−0.004	−3.764	4	0.020
0.5 FA-MS–0.5 FNA-MS	0.016	0.004	0.002	0.011	0.022	8.398	4	0.001
0.5 FA-MS–0.5 UFA-MS	−0.010	0.005	0.002	−0.016	−0.004	−4.673	4	0.009
0.5 FNA-MS–0.5 UFA-MS	−0.026	0.007	0.003	−0.034	−0.018	−8.835	4	0.001
0.5 FNA-MS–0.5 UFNA-MS	−0.008	0.008	0.004	−0.018	0.002	−2.121	4	0.101
0.5 FA-OES–0.5 FNA-OES	0.007	0.005	0.002	0.001	0.013	3.259	4	0.031
0.5 FA-OES–0.5 UFA-OES	−0.006	0.002	0.001	−0.009	−0.003	−5.363	4	0.006
0.5 FNA-OES–0.5 UFA-OES	−0.013	0.004	0.002	−0.018	−0.008	−7.093	4	0.002
0.5 FNA-OES–0.5 UFNA-OES	−0.021	0.005	0.002	−0.028	−0.015	−9.248	4	0.001

Results obtained with the use of the IBM SPSS Statistics software

*FA* filtered but acidified, *FNA* filtered but not acidified, *UFA* unfiltered but acidified, *UFNA* unfiltered but not acidified

The test results of paired samples prove that both sampling and method of analysis influence the boron concentration in the certified samples analysed (Table 9). The differences between the results obtained using the four sampling protocols tested are statistically significant. The results from the ICP-MS method are the most accurate (the analysed value is closer to the true value than with the ICP-OES method). It is necessary to indicate that these differences make a difference of no more than 5 % to the results. It is a lower range than the uncertainty declared by the laboratory for boron analysis. This means that both the ICP-MS and ICP-OES methods can be used for determination of the boron concentration in water.

These results show that the analysis of measurement uncertainty arising from sampling is a very important problem.

## Conclusions

According to the European Standard (EN ISO 5667–3:2012), waters must be filtered on site where the dissolved elements need to be analysed. Membrane filters with a nominal pore size of 0.45 μm should be used. Filtration needs to be done as soon as possible after sample collection. Filtration is not necessary for the determination of the total element concentration. Regardless of the form of the elements determined (total or dissolved), all samples for boron analysis should be preserved by the addition of HNO_3_ to pH 1–2. The same procedure is required for sample preparation for analysis using the ICP-MS and ICP-OES method (EN ISO 17294–2:2004 and EN ISO 11885:2009).

A total of 56 samples were collected during the different desalination processes: ultrafiltration and a double reverse osmosis system connected in series (Tomaszewska and Bodzek 2013a, 2013b). The authors analysed four sampling protocols:

1. Fourteen samples were filtered (through a membrane filter of 0.45 μm) and acidified (using 1 mL ultrapure nitric acid for each 100 mL of samples) (FA)
2. Fourteen samples were unfiltered and not acidified (UFNA)
3. Fourteen samples were filtered but not acidified (FNA)
4. Fourteen samples were unfiltered but acidified (UFA)

All samples were analysed using the ICP-OES and ICP-MS methods.

An analysis of the influence of sample filtration and preservation shows that there is a statistically significant difference between the results obtained using the four sampling protocols analysed (Fig. 2). Also, the method of analysis influences the final results of the boron concentrations in water samples. This difference is also statistically significant (Tables 8 and 9, Fig. 1). Generally, the mean values of the results obtained by ICP-OES are higher than the mean values of the results obtained by ICP-MS. Also, the ranges of concentrations of boron in the samples analysed obtained by ICP-OES are greater than those obtained using ICP-MS, regardless of the method of preparing the samples for analysis (Table 5). It is necessary to indicate that these produce differences of no more than 5 % in the results. It is a lower range than the uncertainty declared by the laboratory for boron analysis. This means that the four sampling protocols tested are correct and can be used during boron analysis. For the determination of the dissolved boron concentration, sample filtration in the field is recommended, whereas for determination of the total boron concentration, filtration should be omitted. Sample acidification meant that the boron in the final acidic solution existed as boric acid which is an undissociated and stable form (Al-Ammar et al. 1999). Both the ICP-MS and ICP-OES method can be used for the determination of boron concentrations in water. The differences in boron concentration obtained using these two methods can be caused by several high-level concentrations in selected whole-water digestates and some matrix effects. The presence of more iron (from 1 to 20 mg/L) than chromium (0.02–1 mg/L) in the samples analysed can influence boron determination. When the iron concentration is high, we can observe a double-joined emission spectrum with an overlapping peak.
